# Draft Genome Sequence of *Rhizopus chinensis* CCTCCM201021, Used for Brewing Traditional Chinese Alcoholic Beverages

**DOI:** 10.1128/genomeA.00195-12

**Published:** 2013-02-28

**Authors:** Dong Wang, Rong Wu, Yan Xu, Ming Li

**Affiliations:** Key Laboratory of Industrial Biotechnology, Ministry of Education, State Key Laboratory of Food Science and Technology, School of Biotechnology, Jiangnan University, Wuxi, China

## Abstract

The filamentous fungus genus *Rhizopus* has traditionally been used for brewing alcoholic beverages and fermented foods in China. The 45,666,236-bp draft genome sequence of *R. chinensis* CCTCCM201021, isolated from the leaven Daqu, was determined, annotated, and analyzed. Analysis of the sequence might provide insight into the properties of this fungus and lead to its further development for industrial applications.

## GENOME ANNOUNCEMENT

In the production of Chinese traditional alcoholic beverages by solid-state fermentation, such as for Chinese rice wine and Chinese liquor, several species of the filamentous fungus genus *Rhizopus* have been widely applied in the brewing processes as microorganisms for saccharification (1, 2). They can secrete large amounts of hydrolytic enzymes, and they play a very important role in converting the starch present in grains into sugars. Furthermore, *Rhizopus* is an important microorganism in the production of various fermentation products (3, 4). However, *Rhizopus* spp. have been poorly described, and our understanding of their mechanisms of growth and metabolism should be improved.

As a saccharifying agent, *Rhizopus chinensis* CCTCCM201021 was isolated from Daqu, a traditional leaven in the production of Chinese liquor (5). Here, we present its draft genome sequence. The genomic DNA of this strain was sequenced with 97-fold coverage using Solexa paired-end and mate-paired sequencing technology. The genome was assembled by Short Oligonucleotide Alignment Program (SOAP)*denovo* (Beijing Genomics Institute [BGI]) (6), which generated 3,281 contigs with an *N*_50_ size of 30.3 kb. The total size of the sequences is 45,666,236 bp, with a G+C content of 36.99%. Gene prediction analysis using the Augustus software program (7) yielded a total of 17,676 predicted protein-coding genes, with 94.3% of them (16,666) encoding proteins longer than 100 amino acids. The average gene length was 1,316 bp. The number of tRNAs predicted by the tRNAscan-SE 1.21 server (8) was 107. The average gene density is one gene per 2.583 kb. The protein-coding sequence occupies 50.89% of the sequenced portion of the genome. An estimated total of 61,241 introns, ranging from 22 to 6,714 nucleotides long, with a mean length of 61 nucleotides, are distributed among 78% of *R. chinensis* genes.

Genome annotation on predicted genes was carried out by BLAST searches against a nonredundant protein sequence database and other databases available online, such as Clusters of Orthologous Groups (COG) and the Kyoto Encyclopedia of Genes and Genomes (KEGG); a total of 74.92% genes were successfully annotated, but only 45.50% genes were identified functionally. In the *R. chinensis* genome, we identified 493 glycoside hydrolase (GH) genes based on the Carbohydrate-Active enZymes (CAZy) database (9). This number was much greater than those in other koji molds, such as *Aspergillus kawachii* (10). Moreover, 168 protease genes and 121 lipase/esterase genes were identified. Comparison with the published genome sequence of *Rhizopus oryzae* (11) revealed that 9.68% of the coding genes (1,711) in the *R. chinensis* genome were unique, but most of them cannot be identified functionally.

Results from the genomic analysis for typical mycotoxin synthesis pathway genes showed that no polyketide synthase (PKS) genes and only a few genes for nonribosomal peptide synthetase (NRPS) and terpenoid metabolism can be found in the *R. chinensis* genome; this suggests that *R. chinensis* might not have any ability to synthesize mycotoxins.

Further in-depth analysis of the *R. chinensis* genome would provide a crucial reference to improve studies about the *Rhizopus* genus, even about the phylum *Zygomycetes*, in which only three fungal genomes have been sequenced completely.

### Nucleotide sequence accession number.

The draft genome sequence of *Rhizopus chinensis* has been deposited at GenBank under the accession no. ANKS00000000.
